# Low-dose aspirin does not improve ovarian stimulation, endometrial response, or pregnancy rates for in vitro fertilization

**DOI:** 10.1186/1743-1050-2-8

**Published:** 2005-05-31

**Authors:** Bradley S Hurst, Jennifer T Bhojwani, Paul B Marshburn, Margaret A Papadakis, Terry A Loeb, Michelle L Matthews

**Affiliations:** 1 Department of Obstetrics and Gynecology, Carolinas Medical Center, 1000 Blythe Blvd, Charlotte, NC 28203, USA

**Keywords:** aspirin, embryo transfer, endometrium, fertilization in vitro, infertility

## Abstract

**Background:**

The purpose of this study is to determine if low-dose aspirin improved ovarian stimulation, endometrial response, or IVF pregnancy rates in our program.

**Methods:**

Retrospective analysis of 316 consecutive IVF cycles from 1995 through 2001. Aspirin 80 mg daily was initiated at the start of luteal leuprolide in 72 cycles. The 244 controls received no aspirin during treatment.

**Results:**

The live birth rate in aspirin users was 29%, slightly lower compared to 41% in the no aspirin control group (p = 0.07). Implantation rates were 21% with aspirin and 30% in the control population (p = 0.01). There was no difference in the maximal endometrial thickness between aspirin and non-aspirin groups. The two groups were similar regarding age, gonadotropin ampules, embryos, number of embryos transferred, prior parity, diagnosis, use of intracytoplasmic sperm injection, and stimulation protocol.

**Conclusion:**

Low-dose aspirin was not beneficial to IVF patients in our program. Aspirin does not enhance endometrial thickness, augment the ovarian response, or improve pregnancy rates.

## Background

Numerous measures have been employed in an attempt to increase implantation and pregnancy rates in assisted reproduction. Aspirin has been utilized as one such potential therapy. This drug has been shown to increase uterine blood flow [1], hence clinicians have postulated that aspirin could improve the receptiveness of the endometrium, thereby increasing implantation and birth rates.

Our institution at one time used aspirin routinely during IVF cycles, based on the work of studies which showed that low-dose aspirin increased implantation and pregnancy rates in women undergoing IVF [2,3]. Contrary data from Urman and co-investigators found no improvement in IVF outcomes with low-dose aspirin [4]. Subsequently, the use of aspirin was stopped in our program early in 2000. Since conflicting results have been reported in the literature, we sought to compare pregnancy rates along with other IVF outcome variables retrospectively in the two groups of women (aspirin vs. non-aspirin) at our institution.

## Methods

This study was a retrospective analysis of 316 consecutive IVF cycles from 1995 – 2001 at Carolinas Medical Center comparing women who were treated with low-dose aspirin versus those who did not receive aspirin treatment. Aspirin was used in all initial cycles from 1995, and excluded from most, but not all cycles beginning early in 2000 at the discretion of the attending physician. Demographic data including age, parity, cycle number, basal FSH, diagnosis, method of stimulation, and use of intracytoplasmic sperm injection was obtained from our database. For the purpose of this study, we divided method of stimulation into GnRH antagonists, long luteal leuprolide, and micro-dose flare. The infertility diagnoses were categorized into male factor, endometriosis, tubal factor, ovulatory dysfunction, unexplained, and other, which included uterine factors and immunological causes. The pregnancy and delivery rates were stable in our program from 1995 to 2001.

Seventy-two aspirin cycles were reviewed along with 244 non-aspirin cycles. For the aspirin cycles, 80 mg of aspirin daily was initiated at the start of down-regulation with luteal leuprolide. Aspirin was started on the first day of leuprolide in microdose flare stimulations. Patients were instructed to continue aspirin until they received the results of their pregnancy tests. The controls received no aspirin at any point during treatment. The outcome measures from the completed cycles were then reviewed. Of interest were the number of gonadotropin ampules used, endometrial thickness, number of eggs fertilized, number of embryos transferred, implantation rate, pregnancy rate, and live birth rate.

### Statistics

The main independent variable was treatment with aspirin (yes/no). Demographic and clinical characteristics for each treatment group were reported and compared with two-tailed t-test, Wilcoxon Rank Sum test, Chi-Square or Fisher's Exact tests, as appropriate. The study outcomes were analyzed in two stages: the first with Chi-Square tests followed by a confirmatory analysis using a regression method generalized estimating equations (GEE). Further analysis of the outcomes assessed their association with aspirin treatment after controlling for other patient and clinical characteristics. The power of the study to determine a difference in pregnancy rates with and without aspirin based on previous studies was approximately 60–72% with an alpha of 0.05 [2,3].

## Results and Discussion

There was no significant difference between age, previous pregnancy, infertility diagnosis, prior IVF, basal FSH, and method of stimulation between the aspirin and non-aspirin groups. (Table 1) More women in the non-aspirin group had been pregnant before (15.9% v. 9.7%) compared to the aspirin group, but this did not achieve statistical significance (p = 0.06).

**Table 1:** Demographic Data

	Aspirin	No aspirin	p
Number patients	72 (23%)	244 (77%)	
Age	34 ± 4	34 ± 4	0.7
Previously pregnant	7 (10%)	39 (16%)	0.06
Diagnosis			
• Unexplained	4 (6%)	12 (5%)	
• Male factor	23 (32%)	51 (21%)	
• Endometriosis	9 (13%)	51 (21%)	
• Tubal factor	13 (18%)	54 (22%)	
• Ovulatory dysfunction	12 (17%)	24 (10%)	
• Other	6 (8%)	17 (7%)	
• Multiple diagnoses	4 (6%)	54 (22%)	
Prior IVF	32%	22%	0.12
Basal FSH (mIU/mL)	7 ± 2	7 ± 8	0.14
Stimulation method			
• Antagonist	0	2 (1%)	
• Long luteal leuprolide	62 (86%)	195 (80%)	
• Flare	8 (11%)	34 (14%)	

Low-dose aspirin did not improve any IVF outcomes analyzed in this study, even though more embryos were transferred to women who used aspirin (p = 0.03) (Table 2). In fact, the pregnancy rate in aspirin users was 48%, slightly lower compared to non-users, 57% (p = 0.18). Clinical pregnancy rates were 45% and 54% for users and non-users, respectively. Live birth rates tended to be lower with aspirin, 29% and 41%, respectively (p = 0.07). Implantation rates were significantly lower in patients who received aspirin, 21% and 30%, respectively (p = 0.01). Maximal endometrial thickness was not improved with aspirin compared to non-aspirin controls (p = 0.26). The percentage of ICSI cycles was similar in each group, as was the number of eggs fertilized.

**Table 2:** Results

	Aspirin	No Aspirin	P
Ampules (75 IU)	42 ± 15	44 ± 17	0.35
Endometrial thickness	12 ± 2	12 ± 2	0.26
ICSI	23 (32%)	67 (28%)	0.46
Oocytes fertilized	9 ± 4	9 ± 6	0.7
Embryos transferred	4 ± 1	3 ± 1	0.03
Pregnancy rate	48%	57%	0.18
Live birth rate	29%	41%	0.07
Implantation rate	21%	30%	0.01

Low-dose aspirin did not benefit IVF patients in our program. Aspirin therapy did not enhance endometrial thickness, augment the ovarian response, or improve pregnancy rates. The demographics were similar between the two groups of patients, with similarities in diagnosis, stimulation protocol, as well as number of ICSI cycles.

Our results conflict with several studies that have shown that aspirin is beneficial for infertility therapy. Rubenstein et al found that aspirin 100 mg starting in the luteal phase of the preceding cycle improved blood flow velocity, ovarian responsiveness, implantation and pregnancy rates in a randomized, controlled trial of 149 patients undergoing IVF compared to 149 placebo-treated controls [2,5]. Weckstein et al also found enhanced uterine blood flow and significantly higher implantation and clinical pregnancy rates with low-dose aspirin in women who had a thin endometrium undergoing embryo transfer from oocyte donation in a randomized controlled study [3].

Interestingly, endometrial thickness was not improved with aspirin. In an prospective, randomized insemination study of women with a thin endometrium undergoing insemination, aspirin improved the percentage of trilaminar endometrium and pregnancy rates from 9 to 18%, but not endometrial thickness or ultrasound flow patterns [6].

Waldenstrom et al randomized 1380 unselected IVF cycles on alternate days to receive aspirin 75 mg or no aspirin starting on the day of embryo transfer and continuing until 18 days after retrieval [7]. In this study, the live birth rate was 27% with aspirin and 23% in the control population, with an odds ratio 1.2 (95% CI 1.0–1.6). A non-controlled study found that IVF outcome was significantly improved when aspirin, heparin, and intravenous immunoglobulin therapy was administered to women with repeat IVF failures and antiphospholipid antibodies, but not to women with negative antiphospholipid antibodies [8]. Other studies have also found a beneficial effect with aspirin/heparin, and aspirin plus prednisolone in IVF patients [9-13]. In vitro studies have shown that aspirin attenuates placental apoptosis, and this could be a possible explanation of how aspirin is beneficial, even in the absence of endometrial or oocyte improvement [14]. Proponents of aspirin consider treatment to be a simple, inexpensive, and harmless means to improve IVF outcomes [7].

However, some studies have shown anticoagulation therapy to be ineffective, and sometimes detrimental, during IVF. A large randomized controlled trial of low-dose aspirin by Urman et al found no difference in implantation or pregnancy rates in patients undergoing ICSI [4]. A higher incidence of ectopic pregnancy was found in the aspirin group. A prospective, randomized, placebo-controlled IVF trial by Stern and colleagues found no benefit with aspirin and heparin for women with prior IVF implantation failure and antiphospholipid or antinuclear antibodies [15]. Another small matched study of women undergoing frozen embryo transfer found an 11% pregnancy rate with aspirin compared to 33% in controls, although the results were not statistically different [16]. Implantation rates were also lower with aspirin therapy, 2.9%, compared to 10.9% in untreated patients in this study. An uncontrolled study of IVF likewise found that outcomes were not improved with aspirin and heparin compared to conventional therapy [17]. Finally, a prospective, randomized, double-blind, placebo-controlled trial of poor responders by Lok et al found no benefit with daily aspirin 80 mg for cancellation rates, total dose of hMG used, number of mature follicles, or number of oocytes retrieved [18]. Furthermore, there was no difference in intraovarian or uterine artery pulsatility index with daily aspirin.

Randomized controlled trials have repeatedly shown that combined aspirin plus heparin improves pregnancy outcomes for women with recurrent pregnancy losses attributed to antiphospholipid antibodies [19,20]. This benefit is also shown in a prospective series [21]. Outcomes are better with aspirin plus heparin therapy than with aspirin alone in most [20,21], but not all studies [22,23]. Aspirin plus corticosteroid therapy, on the other hand, may be harmful. Combined low-dose aspirin plus prednisone increased the risk of preterm birth in two randomized controlled trials [24,25]. With a minimal benefit of aspirin alone for women with recurrent pregnancy losses and antiphospholipid antibodies, it is not surprising that we failed to find a beneficial effect of aspirin therapy in our general IVF population.

In our study, we did not test for uterine blood flow or routinely test for antiphospholipid antibodies. Therefore, we were not able to sub-divide the women in our study into groups that might be more responsive to aspirin. However, an ASRM Practice Committee Report in 1999 concluded that antiphospholipid antibodies do not affect IVF success, and therapy is not justified [26]. Furthermore, we believe that implantation rates, pregnancy rates, and live birth rates are more important indicators of IVF outcome compared to indirect measurements such as endometrial blood flow. In our study, pregnancy, implantation, and live birth rates were higher in the non-aspirin control group.

Another weakness in our study is the six-year period over which our IVF cycles were reviewed. It is possible that subtle differences could bias results in the aspirin and control groups in a retrospective analysis. Additionally, the small study population yields a limited statistical power to detect minor differences in pregnancy outcomes with aspirin. There are actual and sometimes large differences between the two groups of women, which could affect the outcomes. The differencesare not significant, but might be due to the small population studied. There certainly could be minor changes in treatment protocols over that span of time, but our age-related pregnancy and live birth rates remained stable during the years of this study.

Based on the results from our study and the prospective randomized trials by Urman and colleagues [4] and Stern et al [15], aspirin is not beneficial for a general IVF population. Since implantation, pregnancy, and delivery rates are higher for non-aspirin users, our study raises the possibility that aspirin may lower IVF success. A potential fertility reducing effect of aspirin is plausible, since prostaglandins affect ovulation, fertilization, and implantation [27]. Since aspirin inhibits prostaglandin synthesis, implantation could be compromised. Clearly, a larger, prospective randomized study with adequate power would be needed to determine if low-dose aspirin reduced IVF success.

There is some risk associated with aspirin therapy for infertility, although the extent of the risk for a healthy infertility population is unclear. One population based cohort study found that aspirin and nonsteroidal anti-inflammatory agents increased the risk of miscarriage, although a recent meta-analysis showed no increased risk of miscarriage with aspirin [28,29]. Although aspirin does not appear to alter the risk of congenital anomalies, first trimester aspirin consumption may increase the incidence of gastroschisis [30]. Acetylsalicylic acid may reach the uteroplacental circulation and exert antiplatelet effects in the fetus and newborn, although the incidence of neonatal bleeding does not appear to be increased with maternal aspirin [31,32]. However, maternal aspirin may raise the risk of placental abruption and antenatal, intrapartum, and postpartum hemorrhage [32,33]. Additionally, there is at least one reported maternal death due to complications of cerebral hemorrhage in a woman treated with aspirin and heparin after IVF [34]. Although these risks may be small, treatment with aspirin is not justified in the absence of a proven benefit.

## Conclusion

Low-dose aspirin did not enhance endometrial thickness, augment the ovarian response, or improve pregnancy rates in our study. There is no apparent benefit in the routine use of aspirin during IVF cycles, and this practice should be abandoned.

## Competing interests

The author(s) declare that they have no competing interests.

## Authors' contributions

* BSH conceived of the study, participated in the analysis and interpretation of the data, and drafting and revising the manuscript. JTB made substantial contributions to the design and acquisition of data, and drafting the manuscript. PBM, MAP, TAL, and MLM made substantial contributions to the acquisition of data and revising the manuscript. All authors read and approved the final manuscript

## References

[B1] Wada I, Hsu CC, Williams G, Macnamee MC, Brinsden PR (1994). The benefits of low-dose aspirin therapy in women with impaired uterine perfusion during assisted conception. Hum Reprod.

[B2] Rubinstein M, Marazzi A, Polak de Fried E (1999). Low-dose aspirin treatment improves ovarian responsiveness, uterine and ovarian blood flow velocity, implantation, and pregnancy rates in patients undergoing in vitro fertilization: a prospective, randomized, double-blind placebo-controlled assay. Fertil Steril.

[B3] Weckstein LN, Jacobson A, Galen D, Hampton K, Hammel J (1997). Low-dose aspirin for oocyte donation recipients with a thin endometrium: prospective, randomized study. Fertil Steril.

[B4] Urman B, Mercan R, Alatas C, Balaban B, Isiklar A, Nuhoglu A (2000). Low-dose aspirin does not increase implantation rates in patients undergoing intracytoplasmic sperm injection: a prospective randomized study. J Assisted Reprod Genet.

[B5] Polak de Fried E (1999). Errata. Fertil Steril.

[B6] Hsieh YY, Tsai HD, Chang CC, Lo HY, Chen CL (2000). Low-dose aspirin for infertile women with thin endometrium receiving intrauterine insemination: a prospective, randomized study. J Assist Reprod Genet.

[B7] Waldenstrom U, Hellberg D, Nilsson S (2004). Low-dose aspirin in a short regimen as standard treatment in in vitro fertilization: a randomized, prospective study. Fertil Steril.

[B8] Sher G, Zouves C, Feinman M, Maassarani G, Matzner W, Chong P, Ching W (1998). A rational basis for the use of combined heparin/aspirin and IVIG immunotherapy in the treatment of recurrent IVF failure associated with antiphospholipid antibodies. Am J Reprod Immunol.

[B9] Hasegawa I, Hamanoto Y, Suzuki M, Murakawa H, Kurabayshi T, Takakuwa K, Tanaka K (1998). Prednisolone plus low-dose aspirin improves the implantation rate in women with autoimmune conditions who are undergoing in vitro fertilization. Fertil Steril.

[B10] Sher G, Matzner W, Feinman M, Maassarani G, Zouves C, Chong P, Ching W (1998). The selective use of heparin/aspirin therapy, alone or in combination with intravenous immunoglobulin G, in the management of antiphospholipid antibody-positive women undergoing in vitro fertilization. Am J Reprod Immunol.

[B11] Sher G, Maassarani G, Zouves C, Feinman M, Sohn S, Matzner W, Chong P, Ching W (1998). The use of combined heparin/aspirin and immunoglobulin G therapy in the treatment of in vitro fertilization patients with antithyroid antibodies. Am J Reprod Immunol.

[B12] Sher G, Feinman M, Zouves C, Kuttner G, Maassarani G, Salem R, Matzner W, Ching W, Chong P (1994). High fecundity rates following in-vitro fertilization and embryo transfer in antiphospholipid antibody seropositive women treated with heparin and aspirin. Hum Reprod.

[B13] Geva E, Amit A, Lerner-Geva L, Yaron Y, Daniel Y, Schwartz T, Azem F, Yovel I, Lessing JB (2000). Prednisone and aspirin improve pregnancy rate in patients with reproductive failure and autoimmune antibodies: a prospective study. Am J Reprod Immunol.

[B14] Bose P, Black S, Kadyrov M, Weissenborn U, Neulen J, Regan L, Huppertz B (2005). Heparin and aspirin attenuate placental apoptosis in vitro: implications for early pregnancy. Am J Obstet Gynecol.

[B15] Stern C, Chamley L, Norris H, Hale L, Baker HW (2003). A randomized, double-blind, placebo-controlled trial of heparin and aspirin for women with in vitro fertilization implantation failure and antiphospholipid or antinuclear antibodies. Fertil Steril.

[B16] Check JH, Dietterich C, Lurie D, Nazari A, Chuong J (1998). A matched study to determine whether low-dose aspirin without heparin improves pregnancy rates following frozen embryo transfer and/or affects endometrial sonographic parameters. J Assist Reprod Genet.

[B17] Kutteh WH, Yetman DL, Chantilis SJ, Crain J (1997). Effect of antiphospholipid antibodies in women undergoing in-vitro fertilization: role of heparin and aspirin. Hum Reprod.

[B18] Lok IH, Yip SK, Cheung LP, Yin Leung PH, Haines CJ (2004). Adjuvant low-dose aspirin therapy in poor responders undergoing in vitro fertilization: a prospective, randomized, double-blind, placebo-controlled trial. Fertil Steril.

[B19] Triolo G, Ferrante A, Ciccia F, Accardo-Palumbo A, Perino A, Castelli A, Giarratano A, Licata G (2003). Randomized study of subcutaneous low molecular weight heparin plus aspirin versus intravenous immunoglobulin in the treatment of recurrent fetal loss associated with antiphospholipid antibodies. Arthritis Rheum.

[B20] Rai R, Cohen H, Dave M, Regan L (1997). Randomised controlled trial of aspirin and aspirin plus heparin in pregnant women with recurrent miscarriage associated with phospholipids antibodies (or antiphospholipid antibodies). BMJ.

[B21] Kutteh WH (1996). Antiphospholipid antibody-associated recurrent pregnancy loss: treatment with heparin and low-dose aspirin is superior to low-dose aspirin alone. Am J Obstet Gynecol.

[B22] Farquharson RG, Quenby S, Greaves M (2002). Antiphospholipid syndrome in pregnancy: a randomized, controlled trial of treatment. Obstet Gynecol.

[B23] Pattison NS, Chamley LW, Birdsall M, Zanderigo AM, Liddell HS, McDougall J (2000). Does aspirin have a role in improving pregnancy outcome for women with the antiphospholipid syndrome? A randomized controlled trial. Am J Obstet Gynecol.

[B24] Laskin CA, Bombardier C, Hannah ME, Mandel FP, Ritchie JW, Farewell V, Farine D, Spitzer K, Fielding L, Soloninka CA, Yeung M. (1997). Prednisone and aspirin in women with autoantibodies and unexplained recurrent fetal loss. NEJM.

[B25] Silver RK, MacGregor SN, Sholl JS, Hobart JM, Neerhof MG, Ragin A (1993). Comparative trial of prednisone plus aspirin versus aspirin alone in the treatment of anticardiolipin antibody-positive obstetric patients. Am J Obstet Gynecol.

[B26] Practice Committee Report (1999). Antiphospholipid antibodies do not affect IVF success. American Society for Reproductive Medicine.

[B27] Rock JA, Hurst BS (1990). Clinical significance of prostanoid concentration in women with endometriosis. Prog Clin Biol Res.

[B28] Li DK, Liu L, Odouli R (2003). Exposure to non-steroidal anti-inflammatory drugs during pregnancy and risk of miscarriage: population based cohort study. BMJ.

[B29] Kozer E, Nikfar S, Costei A, Boskovic R, Nulman I, Koren G (2002). Aspirin consumption during the first trimester of pregnancy and congenital anomalies: a meta-analysis. Am J Obstet Gynecol.

[B30] Kozer E, Costei AM, Boskovic R, Nulman I, Nikfar S, Koren G (2003). Effects of aspirin consumption during pregnancy on pregnancy outcomes: meta-analysis. Devel Reprod Toxicology.

[B31] Leonhardt A, Bernert S, Watzer B, Schmitz-Zeigler G, Seyberth HW (2003). Low-dose aspirin in pregnancy: maternal and neonatal aspirin concentrations and neonatal prostanoid formation. Pediatrics.

[B32] Sibai BM, Caritis SN, Thom E, Klebanoff M, NcNellis D, Rocco L, Paul RH, Romero R, Witter F, Rosen M (1993). Prevention of preeclampsia with low-dose aspirin in healthy, nulliparous pregnant women. The National Institute of Child Health and Human Development Network of Maternal-Fetal Medicine Units. NEJM.

[B33] Golding J (1998). A randomized trial of low dose aspirin for primiparae in pregnancy. The Jamaica Low Dose Aspirin Study Group. Br J Obstet Gynaecol.

[B34] Centers for Disease Control and Prevention (1998). Pregnancy-related death associated with heparin and aspirin treatment for infertility, 1996. JAMA.

